# Temporal and spatial characteristics of the area at risk investigated using computed tomography and T1-weighted magnetic resonance imaging

**DOI:** 10.1186/1532-429X-17-S1-P154

**Published:** 2015-02-03

**Authors:** Jesper van der Pals, Sophia Hammer-Hansen, Sonia Nielles-Vallespin, Peter Kellman, Joni Taylor, Shawn Kozlov, Li-Yueh Hsu, Marcus Y Chen, Andrew E Arai

**Affiliations:** National Institutes of Health, Bethesda, MD USA

## Background

Cardiovascular magnetic resonance (CMR) imaging can measure the myocardial area at risk (AAR), but the technique has received criticism for inadequate validation. CMR commonly depicts an AAR that is wider than the infarct, which in turn would require a lateral perfusion gradient within the AAR. We investigated the presence of a lateral perfusion gradient within the AAR and validated CMR measures of AAR against 3 independent reference standards of high quality.

## Methods

Computed tomography (CT) perfusion imaging, microsphere blood flow analysis, T1-weighted 3T CMR, and fluorescent microparticle pathology were used to investigate the AAR in a canine model (n=10) of ischemia and reperfusion.

## Results

AAR size by CMR correlated well with CT (R^2^=0.80), microsphere blood flow (R^2^=0.80), and pathology (R^2^=0.74) with good limits of agreement (-0.79±4.02 % of the left ventricular mass (LVM) versus CT; -1.49±4.04 %LVM versus blood flow and -1.01±4.18 %LVM versus pathology). The lateral portion of the AAR had higher perfusion than the core of the AAR by CT perfusion imaging (40.7±11.8 vs 25.2±17.7 Hounsfield units, p=0.0008) and microsphere blood flow (0.11±0.04 vs 0.05±0.02 ml/g/min, lateral vs core, p=0.001). The transmural extent of MI was lower in the lateral portion of the AAR than the core (28.2±10.2 vs 17.4±8.4 % of the wall, p = 0.001).

## Conclusions

T1-weighted CMR accurately quantifies size of the AAR with excellent agreement compared to 3 independent reference standards. A lateral perfusion gradient results in lower transmural extent of infarction at the edges of the AAR compared with the core.

## Funding

The project described was funded by the Division of Intramural Research, National Heart, Lung and Blood Institute, National Institutes of Health.

**Figure 1:**
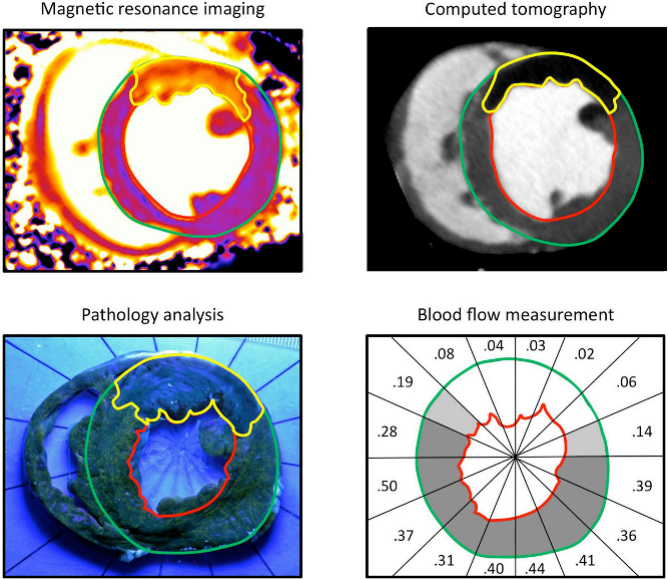
Representative examples of area at risk imaging modalities.

